# Biogeography and Photosynthetic Biomass of Arctic Marine Pico-Eukaroytes during Summer of the Record Sea Ice Minimum 2012

**DOI:** 10.1371/journal.pone.0148512

**Published:** 2016-02-19

**Authors:** Katja Metfies, Wilken-Jon von Appen, Estelle Kilias, Anja Nicolaus, Eva-Maria Nöthig

**Affiliations:** 1 Department of Polar Biological Oceanography, Division of Biosciences, Alfred-Wegener Institute - Helmholtz Centre for Polar and Marine Research, Bremerhaven, Germany; 2 Physical Oceanography of the Polar Seas, Division of Climate Sciences, Alfred-Wegener Institute - Helmholtz Centre for Polar and Marine Research, Bremerhaven, Germany; Auckland University of Technology, NEW ZEALAND

## Abstract

Information on recent photosynthetic biomass distribution and biogeography of Arctic marine pico-eukaryotes (0.2–3 μm) is needed to better understand consequences of environmental change for Arctic marine ecosystems. We analysed pico-eukaryote biomass and community composition in Fram Strait and large parts of the Central Arctic Ocean (Nansen Basin, Amundsen Basin) using chlorophyll *a* (Chl *a*) measurements, automated ribosomal intergenic spacer analysis (ARISA) and 454-pyrosequencing. Samples were collected during summer 2012, the year with the most recent record sea ice minimum. Chl *a* concentrations were highest in eastern Fram Strait and pico-plankton accounted for 60–90% of Chl *a* biomass during the observation period. ARISA-patterns and 454-pyrosequencing revealed that pico-eukaryote distribution is closely related to water mass distribution in the euphotic zone of the Arctic Ocean. Phaeocystaceae, *Micromonas* sp., Dinophyceae and Syndiniales constitute a high proportion of sequence reads, while sequence abundance of autotrophic Phaeocystaceae and mixotrophic *Micromonas* sp. was inversely correlated. Highest sequence abundances of Phaeocystaceae were observed in the warm Atlantic Waters in Fram Strait, while *Micromonas* sp. dominated the abundant biosphere in the arctic halocline. Our results are of particular interest considering existing hypotheses that environmental conditions in Nansen Basin might become more similar to the current conditions in Fram Strait. We propose that in response, biodiversity and biomass of pico-eukaryotes in Nansen Basin could resemble those currently observed in Fram Strait in the future. This would significantly alter biogeochemical cycles in a large part of the Central Arctic Ocean.

## Introduction

Pico-eukaryotes (0.2–2 μm) are important constituents of marine ecosystems. They are known to be ubiquitous in surface waters of the oceans and dominate protist assemblages of oligotrophic waters [1]. Pico-eukaryotes are well adapted to harsh polar environmental conditions and dominate Arctic pelagic phytoplankton communities for most of the year [2, 3]. Investigations carried out in the early 1990s revealed that small phytoplankton (<5 μm) accounted for up to 60–90% of total Chl *a* biomass in areas with high ice-coverage and low phytoplankton production [4, 5]. However, factors that shape community structure and spatial distribution, i.e. biogeography of pico-eukaryotes, are not well understood [6].

Currently, some parts of the Arctic system are undergoing rapid change, while others do not change qualitatively. Air temperatures in the Arctic are rising twice as fast as elsewhere on the globe [7]. The extent, thickness and age of sea ice are decreasing [8] such that a considerable part of the Eurasian Arctic below 85°N is ice free in summer. The remaining sea ice cover in the Arctic is thinner than two decades ago. Recently, at least 50% of the sea ice cover was composed of first-year ice, while the proportion of multi-year sea ice older than 4 years was less than 10% [9]. Light penetration through first-year ice is significantly higher than through multi-year ice [10]. In contrast, stratification in the Arctic halocline in the upper 50–100 m of the water column does not undergo multi-decadal changes and observed variations in the total freshwater stored in the upper Arctic Ocean are instead associated with decadal variability [11]. However, if the sea ice volume decline continues, a point may be reached in the future when not enough sea ice is advected into the Nansen Basin for halocline formation to occur and a regime shift to deep convection would occur (B. Rudels, personal communication 2015).

It is expected that increased light availability due to the decline of Arctic sea ice will positively impact primary productivity in and under the Arctic sea ice. An effect of this might be high diatom biomass and growth rates, however, this is only expected over the nutrient-rich shelf areas [12]. Other studies predict a gradual shift toward small-sized primary producers in nutrient diminished surface waters of a warmer ocean [13]. Either way, changes in the composition of primary producers such as pico-eukaryotes will strongly impact ecosystem production and carbon export in the Arctic Ocean. Thus, studying the recent community structure, biogeography and biomass of pico-eukaryotes in the Arctic Ocean is an important task and will contribute to a better understanding of carbon fluxes in this area at present and in the future.

A better understanding of pico-eukaryote biogeography requires investigating species distributions in relation to the physical properties of the water column. The Arctic marine environment is composed of distinct water masses that are characterized by differences in salinity, temperature, stratification and nutrient concentration. Based on their small cell size and low sinking rates, mesoscale distribution of pico-eukaryotes is mainly determined by passive lateral advection and vertical mixing in the water column [14]. Thus, elucidating the impact of physical oceanographic factors on the composition and distribution of Arctic Ocean pico-eukaryote communities is an important task in the light of expected ecosystem shifts in response to climate change.

Past studies on Arctic pico-eukaryotes mainly analyzed samples collected in the Canadian Arctic south of 80°N at relatively low spatial resolution [15–17]. Studies from the Canadian archipelago report that a pico-eukaryote assemblage was closely associated with water mass origin [18]. In contrast, information on the impact of the Arctic Ocean circulation on the biogeographical patterns of pico-eukaryote communities including the area north 80°N is very limited. Here we present a study covering large parts of the Central Arctic Ocean to elucidate the Chl *a* biomass distribution and biogeographic patterns of pico-eukaryote communities in relation to ambient water masses and sea ice coverage during 2012, the year with the most recent record sea ice minimum [19].

## Material and Methods

### Sampling

**Fig 1:**
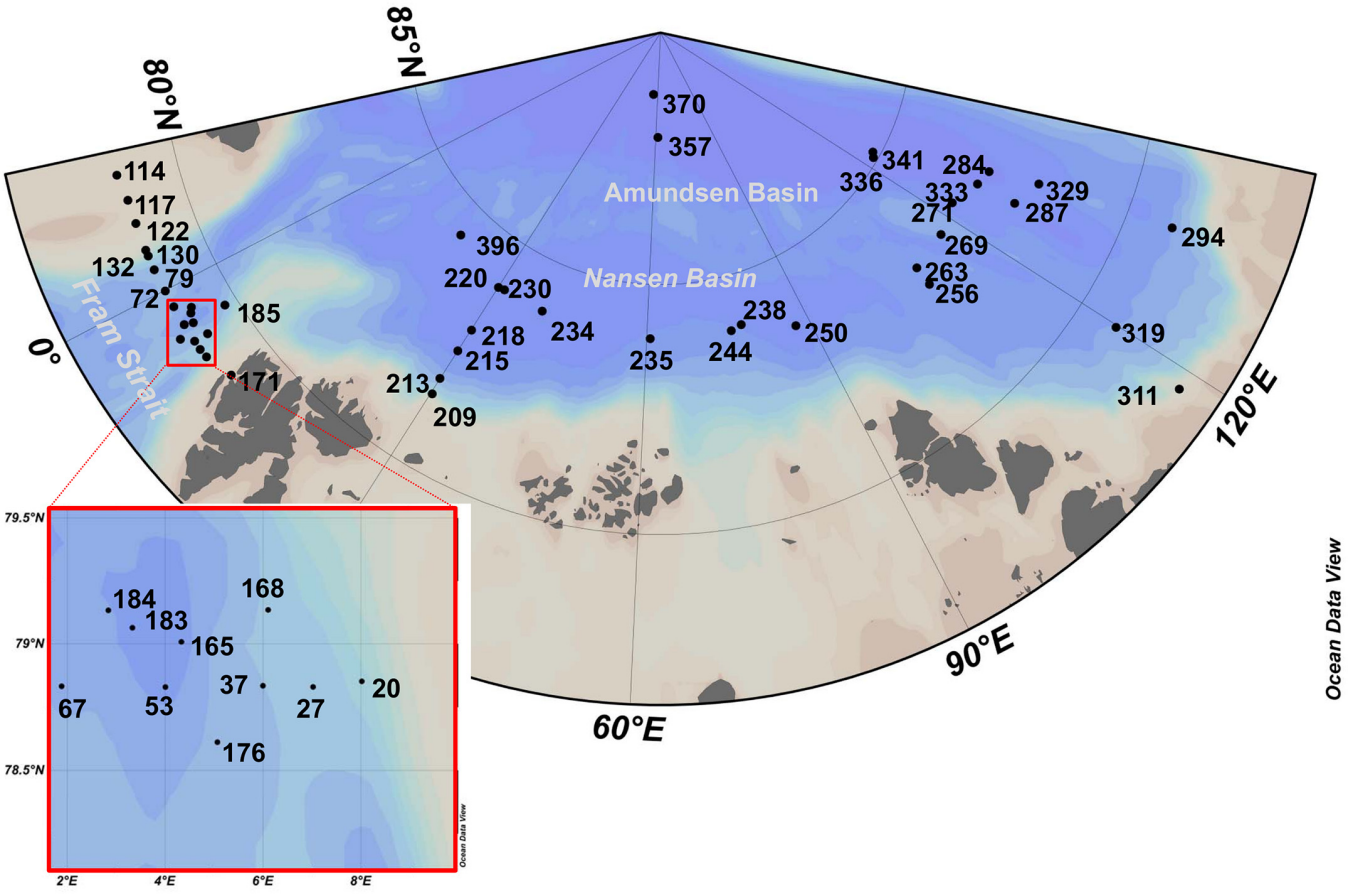
Map of the study area including the sampling sites that were occupied during expedition PS80 /ARK-XXVII/1-3 of RV Polarstern.

The samples were collected during all three cruise legs of RV Polarstern cruise ARK-XXVII (PS80) to the Arctic Ocean (Fig 1). The cruise began in June, 2012 in the eastern Fram Strait (station 20) and ended in September of the same year in the Nansen Basin (station 396) (Table 1). Sample number reflects the order of sampling and the cruise track. Stations were in international waters and in the exclusive economic zones of Denmark, Norway and Russia. No permission was required for sampling in international waters and diplomatic permissions for sampling in the exclusive economic zones were obtained from the responsible authorities (Ministry of Foreign Affairs, Denmark; Directorate of Fisheries-Resource Management Department, Norway; Ministry of Foreign Affairs, Russia). The field study did not involve endangered or protected species.

**Table 1:** Environmental parameter of the water column at the chlorophyll *a* maximum of sampling sites^a^.

Station	Sampling date	Ice concentration [%]	Water temperature [°C]	Salinity	NO^-^_3_ [μmol L^-1^]	Silicate [μmol L_-1_]	PO^-2^_4_ [μmol L^-1^]
**PS80/79**	30.06.2012	95	-1.69	33.75	5.67	2.74	0.58
**PS80/114**	06.07.2012	99	-1.57	31.55	NA	NA	NA
**PS80/117**	06.07.2012	57	-1.55	31.72	0.83	1.94	0.55
**PS80/122**	07.07.2012	86	-1.46	31.33	NA	NA	NA
**PS80/130**	08.07.2012	91	-1.58	32.02	NA	NA	NA
**PS80/132**	09.07.2012	100	-1.49	32.48	4.10	7.56	0.64
**Average Fram Strait, E of 0°**		**88**	**-1.56**	**32.14**	**3.53**	**4.08**	**0.59**
**PS80/20**	20.06.2012	0	5.25	35.07	3.58	1.88	0.30
**PS80/27**	21.06.2012	0	4.37	35.11	10.12	4.38	0.71
**PS80/37**	22.06.2012	0	4.82	35.11	4.14	4.04	0.39
**PS80/53**	25.06.2012	8	4.98	34.96	9.44	3.97	0.71
**PS80/67**	27.06.2012	59	4.10	35.03	4.04	3.71	0.42
**PS80/72**	28.06.2012	79	2.80	34.51	2.61	4.15	0.42
**PS80/165**	16.07.2012	0	5.02	35.01	3.24	3.72	0.39
**PS80/168**	17.07.2012	0	5.91	34.94	3.18	2.46	0.37
**PS80/171**	18.07.2012	0	3.04	34.56	NA	NA	NA
**PS80/176**	20.07.2012	0	6.07	35.04	3.36	3.80	0.40
**PS80/183**	22.07.2012	0	5.00	34.93	NA	NA	NA
**PS80/184**	22.07.2012	0	6.04	35.04	10.19	5.12	0.74
**PS80/185**	23.07.2012	32	4.69	34.90	0.17	3.01	0.21
**AverageFram Strait, W of 0°**		**14**	**4.78**	**34.94**	**4.91**	**3.66**	**0.46**
**PS80/209**	06.08.2012	0	-0.04	34.27	5.25	2.41	0.49
**PS80/213**	06.08.2012	0	-1.16	33.70	0.72	1.25	0.20
**PS80/215**	07.08.2012	52	-1.11	33.26	0.46	0.99	0.17
**PS80/218**	07.08.2012	91	-1.74	34.25	5.57	1.90	0.45
**PS80/220**	08.08.2012	96	-1.78	34.19	4.21	1.37	0.38
**PS80/230**	11.08.2012	96	-1.77	34.18	NA	NA	NA
**PS80/234**	12.08.2012	94	-1.52	34.04	3.95	1.36	0.35
**PS80/235**	13.08.2012	98	-1.68	34.18	7.57	2.97	0.54
**PS80/238**	14.08.2012	100	-1.72	34.15	4.79	1.65	0.41
**PS80/244**	16.08.2012	90	-1.58	34.17	6.64	2.67	0.51
**PS80/250**	18.08.2012	73	-1.68	34.13	4.61	1.69	0.41
**AverageNansen Basin**		**72**	**-1.43**	**34.05**	**4.38**	**1.83**	**0.39**
**PS80/256**	20.08.2012	93	-1.68	33.74	1.13	1.23	0.22
**PS80/263**	22.08.2012	94	-1.67	33.09	2.21	1.68	0.28
**PS80/269**	23.08.2012	82	-1.62	33.19	2.49	2.39	0.32
**PS80/271**	24.08.2012	53	-1.39	31.43	2.27	4.54	0.34
**PS80/284**	26.08.2012	95	-1.55	31.18	0.59	3.62	0.24
**PS80/287**	27.08.2012	10	-1.37	31.38	1.45	4.44	0.30
**PS80/294**	29.08.2012	0	-1.46	32.04	1.81	4.01	0.30
**PS80/311**	01.09.2012	0	-0.27	32.10	0.21	1.28	0.19
**PS80/319**	02.09.2012	0	-1.46	31.84	0.45	2.75	0.22
**PS80/329**	05.09.2012	49	-1.48	31.04	0.90	3.42	0.28
**PS80/333**	06.09.2012	0	-1.50	31.04	1.00	4.65	0.28
**PS80/336**	07.09.2012	80	-1.55	31.47	1.71	4.68	0.34
**PS80/341**	09.09.2012	66	-1.54	29.97	0.54	4.69	0.25
**PS80/357**	19.09.2012	100	-1.80	33.11	1.00	1.52	0.23
**PS80/370**	23.09.2012	100	-1.79	32.93	1.68	2.23	0.28
**PS80/396**	29.09.2012	100	-1.79	32.78	2.18	1.15	0.28
**AverageAmundsen Basin**		**58**	**-1.49**	**32.02**	**1.35**	**3.02**	**0.27**

^a^All nutrient data are available in the PANGAEA database (www.pangaea.de).

Water samples were taken at 46 stations located in Fram Strait and the Central Arctic Ocean. The cruise track in the Central Arctic Ocean was mainly in close proximity to the ice edge. Sampling was carried out with a rosette sampler equipped with 24 Niskin bottles (12 L per bottle) and sensors for Chl *a* fluorescence, temperature and salinity (CTD). Samples were taken during the upcasts at the vertical maximum of Chl *a* fluorescence determined during the downcasts. The sampling depths varied between 10–50 m. Samples were collected in Fram Strait in June/July, while sampling in the Central Arctic Ocean took place in August/September. Two-liter subsamples were taken in PVC bottles from the Niskins. Pico-plankton cells for both Chl *a* measurements and molecular analyses were collected by sequential filtration of one water sample through three different mesh sizes (10, 3, and 0.4 μm) on 45 mm diameter Isopore Membrane Filters at 200 mbar using a Millipore Sterifil filtration system (Millipore, USA). Flow cytometry data indicated that cyanobacteria contributed only ~1% of the cells to the pico-plankton size fraction (Ilka Peeken personal communication). Therefore, cells containing Chl *a* collected on the 0.4 μm filter represent mainly the pico-eukaryote fraction. Filters were stored in Eppendorf tubes (Eppendorf, Germany) at -80°C until further processing in the laboratory.

### Measurement of chlorophyll *a*

The filters were extracted in 90% acetone overnight and analyzed with a fluorometer (Turner Design, USA) slightly modified to the methods described in [20] and [21]. Calibration of the fluorometer was carried out with standard solutions of Chl *a* (Sigma, Germany). Total Chl *a* concentrations are calculated by adding the Chl *a* concentrationsdetermined for the different size fractions.

### DNA isolation

Genomic DNA was extracted from cells collected on filters with 0.4 μm pore size. DNA extraction was carried out with E.Z.N.A TM SP Plant DNA Kit Dry Specimen Protocol (Omega Bio-Tek, USA) following the manufacturer’s protocol. The extracts were stored at -20°C until analysis.

### ARISA

The intergenic spacer region of the ribosomal operon was amplified from the genomic DNA using primers 1528F (5′-GTA GGT GAA CCT GCA GAA GGA TCA-3′ [22]) and ITS2 (5′-GCT GCG TTC TTC ATC GAT GC-3′ [23]). Primer 1528F was fluorescently labeled with 6-FAM. The PCR-amplifications were performed in a 20 μL volume in a thermal cycler (Eppendorf, Germany) using 1× HotMasterTaq buffer containing Mg^2+^, 2.5 mM (5′Prime); 0.02 U HotMaster Taq polymerase (5’Prime, Germany); 0.4 mg mL^-1^ BSA; 0.8 mM (each) dNTP (Eppendorf, Germany); 0.2 μM of each primer and 1μL of template DNA (20 ng μL^-1^). The amplification was based on 35 cycles, consisting of 94°C for 45 sec, 55°C for 1 min and 72°C for 3 min, proceeded by 3 min denaturation at 94°C and followed by a final extension of 10 min at 72°C. The size of the PCR fragments was determined by analysis with a capillary sequencer (ABI 3130XL, Applied Biosystems, Germany). The ARISA analysis was carried out in triplicate for each sample. The quality control and analysis of the raw data were carried out with the GeneMapper v4.0 (Applied Biosystems, Germany) software. This included the application of a threshold of 50 base pairs (bp) for counting peaks in order to exclude false positive peaks originating from primers or by the formation of primer dimers.

### Statistical analyses

In an ARISA-analysis the community is characterized by its community profile, which is based on the composition (presence/absence) of differently sized DNA fragments. The DNA fragments are a result of the amplification of the internal transcribed spacer region of the ribosomal operon, which displays a high degree of taxon-related variability in its length. In this study, presence/absence matrices reflecting the community profiles of the samples were generated by binning the quality controlled data obtained after size separation with the capillary sequencer using the “Interactive Binner” [24]. Differences in the ARISA community profiles were estimated by calculating the Jaccard index. The Jaccard index is a statistical method used for comparing the similarity and diversity of sample sets [25]. It measures the similarity between samples. The result of the analysis is a distance matrix of the samples in the data set. The resulting distances were visualized by multidimensional scaling (MDS) with the vegan software package (http://r-forge.r-project.org/projects/vegan/). Groups in the MDS plot were determined *à priori* based on automated clustering using the hclust function and the agglomeration method ward in R. The significance of the grouping was tested by analyzing the similarity between the groups with an ANOSIM analysis [26]. ANOSIM is a multivariate, non-parametric statistical method used for comparing community compositions among groups of samples. Correlations of environmental parameters and molecular data, and similarities between ARISA and 454-pyrosequencing were evaluated with a Mantel-Test. All statistical analyses were carried out within R (R Development Core Team (*2011)*, *URL* http://www.R-project.org/). R-scripts for the “Interactive Binner” (S1 File) and *à priori* grouping of ARISA profiles (S2 File) are provided as supplements to this publication.

### 454-pyrosequencing

For 454-Sequencing, a ~670 bp fragment of the 18S rDNA containing the hypervariable V4 region was amplified with the primer set 528F (GCG GTA ATT CCA GCT CCA A) and 1055R (ACG GCC ATG CAC CAC CAC CCA T) [27]. All PCRs had a final volume of 50 μL and contained 0.02 U HotMaster Taq polymerase (5’Prime), the 10-fold polymerase buffer according to manufacturer’s specification, 0.4 mg mL^-1^ BSA, 0.8 mM (each) dNTP (Eppendorf, Germany), 0.2 μmol L^-1^ of each Primer and 1μL of template DNA. PCR amplification was performed in a thermal cycler (Eppendorf, Germany) with an initial denaturation (94°C, 5 min) followed by 35 cycles of denaturation (94°C, 1 min), annealing (58°C, 2 min), and extension (72°C, 2 min) with a single final extension (72°C, 10 min). The PCR products were purified with the Mini Elute PCR Purification kit (Qiagen, Germany). Finally, the sequencing of the amplicon was performed by GATC Biotech (Germany), using a 454 GS FLX Titanium sequencer (Roche, Germany). Raw sequences had an approximate length of 310 bp. Sequences generated in this study have been deposited at the European Nucleotide Archive (ENA) under Accession PRJEB1449.

### Data analysis 454-pyrosequencing

Raw sequence reads were processed to obtain high quality reads. The primer set used in this study amplifies a PCR product of ~500 bp including the V4-region of the 18S rRNA gene. The forward primer 528F, used for the sequencing, attaches approximately 25 bp upstream of the V4 region, which has an approximate length of 230 bp [28]. Thus, reads with a length under 300 bp were excluded from further analysis to ensure including the complete V4 region in the analysis and to remove short reads. Unusually long reads, greater than the expected amplicon size (>670 bp), and reads with more than one uncertain base (N) were also removed from the analyses. Chimeric sequences in the remaining data set were eliminated from further analyses based on an assessment using the software UCHIME 4.2 [29]. The resulting high quality reads of all samples were grouped into operational taxonomic units (OTUs) at the 97% similarity level using software Lasergene 10 (DNASTAR, USA), which is using the farthest neighbour method for clustering of sequences. Reads not starting with the forward primer were manually removed. Consensus sequences of each OTU were generated using the software Lasergene 10, representing the order of the most frequent bases in an OTU. This approach reduced the number of sequences and attenuated the influence of sequencing errors and uncertain bases. The 97% similarity level has shown to be the most suitable to reproduce original eukaryotic diversity [30] and also has the effect of bracing most sequencing errors [31]. Furthermore, known intragenomic small subunit SSU polymorphism levels can range to 2.9% in dinoflagellate species [32]. OTUs comprised of only one sequence (singletons) were removed. The consensus sequences were aligned using the software HMMER 2.3.2 [33]. Subsequently, taxonomical affiliation was determined by placing the consensus sequences into a reference tree containing about 1,200 high quality sequences of Eukarya from the SILVA reference database (SSU Ref 108) using the software pplacer 1.0 [34]. A phylogenetic likelihood of 85% was used as a threshold for taxonomic annotation of the sequences. The compiled reference database is available on request in ARB-format. OTUs assigned to fungi and metazoans were excluded from further analysis.

## Results and Discussion

### Environmental parameters

In August 2012, a strong storm reinforced the melting of sea ice in the Central Arctic Ocean, resulting in a record sea ice minimum in September 2012 [19]. During the observation period sea ice concentrations varied in the area. Average sea ice concentrations were highest in Nansen Basin (72%) and lowest in eastern Fram Strait (14%), where 75% of all stations were ice-free (Table 1).

**Fig 2:**
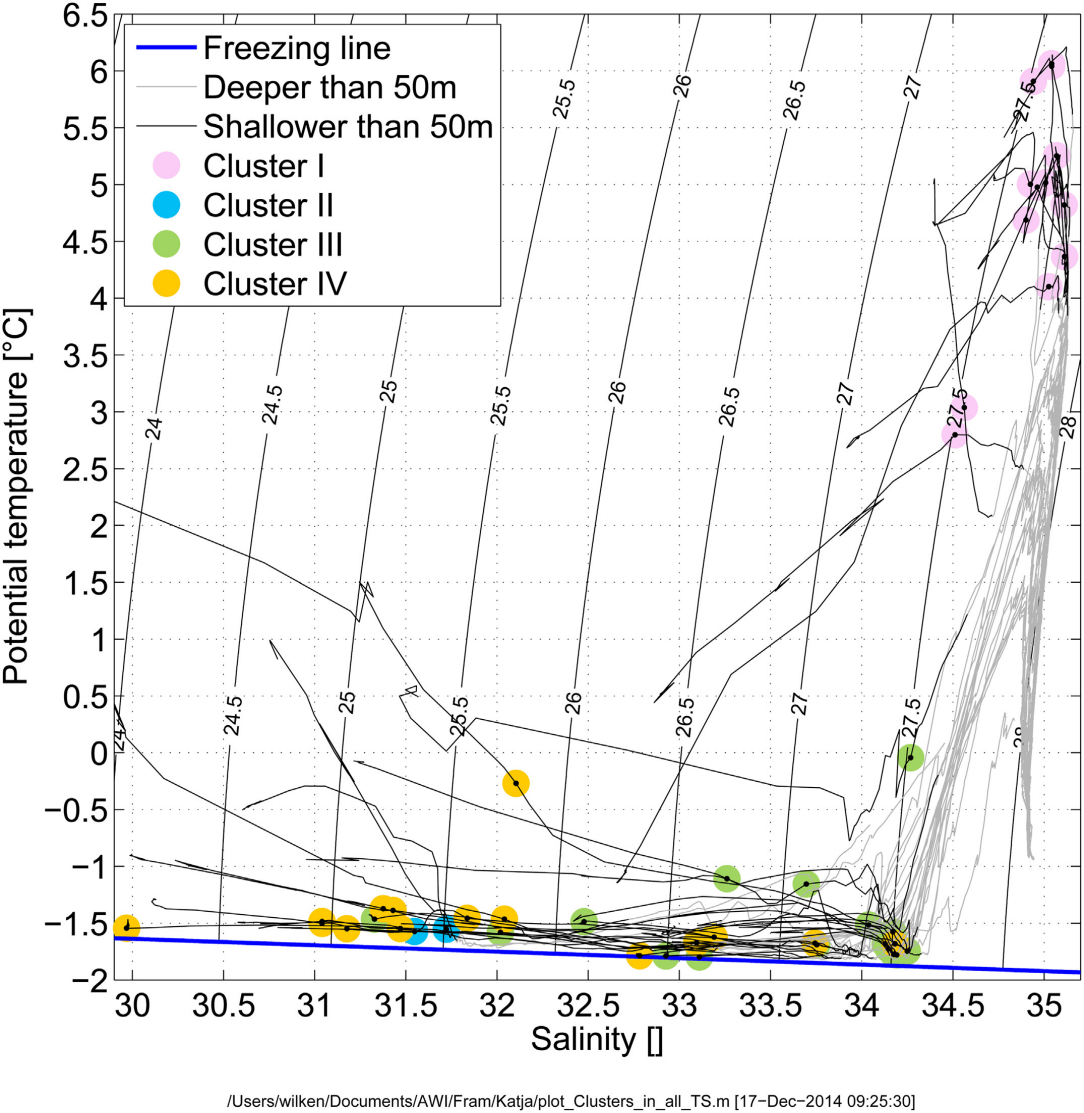
Temperature-salinity diagram of the CTD stations. Profiles shallower than 50 m are shown as black lines and profiles deeper than 50 m as gray lines. The biological samples are marked where they were taken in the water column and named according to the labelling in the metaMDS plot from Fig 4. The surface freezing line is shown in blue and the isopycnals in black.

In order to elucidate the impact of sea ice coverage and different water masses on Arctic pico-plankton Chl *a* biomass and pico-eukaryote biogeography it is necessary to understand the water mass properties in the observation area that includes Fram Strait and large parts of the Eurasian Arctic Ocean (Nansen Basin and Amundsen Basin). Warm Atlantic Water flows through eastern Fram Strait and the Barents Sea into the Arctic to form a cyclonic boundary current along the Arctic Ocean's perimeter [35]. North of Svalbard, it interacts with the sea ice formed during wintertime to produce a layer of fresher and colder water overlying the warm Atlantic boundary current [36]. This Arctic halocline covers the upper 50–100 m of the Arctic. It is characterized by temperatures within a few tenths of a degree Celsius of the freezing line and a wide variation in salinity. The strong vertical salinity gradient leads to a stratification (density difference between 10 and 50 m at the stations in the halocline of 0.5–3 kg m^-3^) and the near complete inhibition of mixing between waters in the euphotic zone with non-nutrient depleted waters below. The western side of Fram Strait is an export region for water and sea ice from the Arctic Ocean to the Nordic Seas [37]. Waters in western Fram Strait have a significant Pacific Water contribution (water having entered the Arctic through Bering Strait), but reflect the halocline properties of the Arctic Ocean and its associated stratification [38]. The water mass characteristics observed in this study at the sampling depth reflect the situation previously described. A significant Pacific Water contribution was observed at stations in western Fram Strait (~10% at stations 72 and 79 and ~30–50% at stations 117 and 132 further to the west) as determined from the nitrate to phosphate ratio [38]. The other sampling locations fall into two distinct cases with a few outliers discussed in detail below. In the TS diagram (Fig 2) this corresponds to a cluster near the highest temperatures and salinities close to 35 ("Atlantic inflow") and to another cluster that follows the freezing line of sea water ("Arctic halocline"). The inflow of warm Atlantic Water to the Arctic is still at the surface in the eastern Fram Strait [39]. Compared to the Central Arctic, the stratification was relatively low in eastern Fram Strait with a density difference between 10 and 50 m of 0.05–0.5 kg m^-3^. This is conducive to mixing of the water in the euphotic zone with the waters below and therefore nutrients can be resupplied to the euphotic zone during the growth period. Furthermore, the eastern Fram Strait under influence of the warm Atlantic Water is ice-free year-round (Table 1).

Nutrient concentrations (PO^2-^_4;_ NO^-^_3_; Si) varied during the observation period. In eastern Fram Strait and Nansen Basin phosphate and nitrate concentrations were in a similar range, but significantly higher than in Amundsen Basin (Table 1). The significance of the differences between nutrient concentration in Nansen Basin versus Amundsen is reflected by p-values (t-test) for PO^2-^_4_ = 0.015, NO^-^_3_ = 0.002, and Si = 0.007. Higher nutrient concentrations in Nansen Basin compared to Amundsen Basin are likely related to the inflow of Atlantic Water to the Nansen Basin. The surface waters in the Amundsen Basin have been in the Arctic halocline for longer and were nutrient depleted in that time period. Additionally they contained a significant fraction of nutrient-poor water from the Siberian shelves. Polar Water of the East Greenland Current (EGC) displayed highest phosphate and silicate concentrations, while the nitrate concentration was in a similar range as observed in eastern Fram Strait. The sample located in Polar Water over the Greenland Shelf (117) was nitrate limited, while also having the highestobserved phosphate concentration observed in this study. Stations occupied during ARK-XXVII that are located neither in the warm Atlantic inflow nor the Arctic halocline, are described in the following: Stations on the Greenland shelf (114 and 117) are influenced by continental runoff from Greenland. The transition between Atlantic waters and polar outflow waters in Fram Strait is sampled by station 72, whose TS falls in between the two end members (Fig 2). The brackish water in Kings Bay, the fjord on which Ny-Ålesund is located, was sampled at station 171. The three southern stations (209, 213 and 215) of the section north of Svalbard are in the halocline formation region. Station 209, the one closest to the coast, is also affected by continental runoff and local near surface warming. Finally, station 311 is on the Laptev Sea shelf-break and affected by the riverine discharge into the Kara and Laptev Seas. Environmental data described in this study can be retrieved from the PANGAEA database:

physical oceanography ARK-XXVII/1:http://doi.pangaea.de/10.1594/PANGAEA.801791;

physical oceanography ARK-XXVII/2: http://doi.pangaea.de/10.1594/PANGAEA.800427;

physical oceanography ARK-XXVII/3: http://doi.pangaea.de/10.1594/PANGAEA.802904; nutrients: http://doi.pangaea.de/10.1594/PANGAEA.834081

### Total chlorophyll *a* biomass

**Fig 3:**
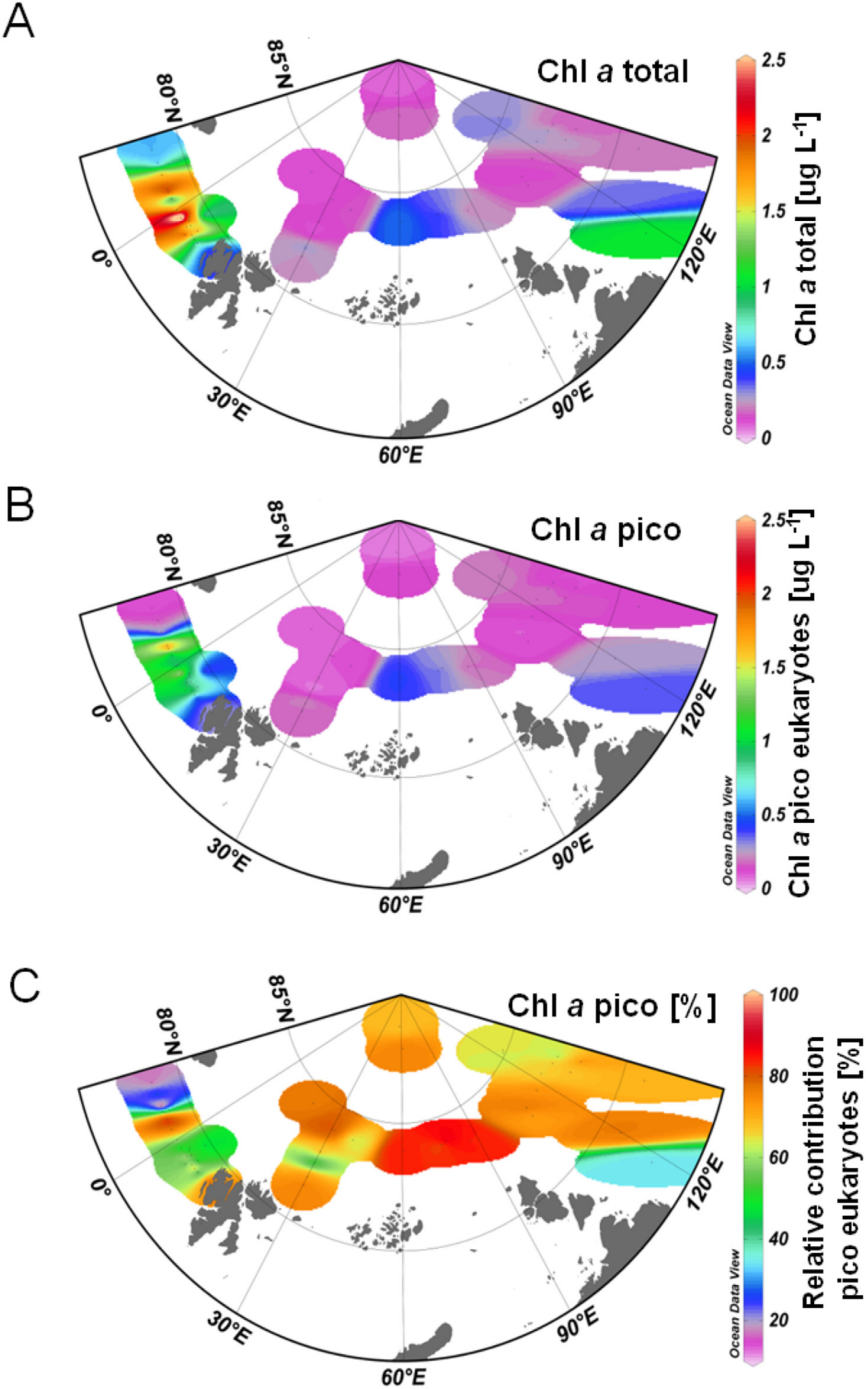
Diagram illustrating the spatial distribution of chlorophyll *a* biomass in the observation area in Fram Strait, Nansen Basin and Amundsen Basin. Data are interpolated based on point measurements and do not reflect measurements in between the sampling sites. A: Spatial distribution of total chlorophyll *a* biomass including micro-, nano-, and pico-plankton. B: Spatial distribution of pico-eukaryotic chlorophyll *a* biomass. C: Relative contribution of pico-eukaryotic chlorophyll *a* biomass to total chlorophyll *a* biomass.

Chl *a* concentrations in the Chl *a* maximum of the fractions <3 μm and >3 μm were measured and summed as an index for total Chl *a* phytoplankton biomass. The Chl *a* maximum in the Central Arctic Ocean was always located in the upper halocline and coincided with the depth of the highest observed temperatures in the water column. The seawater above the Chl *a* maximum was a mixture of sea waterand meltwater (T = freezing temperature, S ≈ 32) that was formed by melting sea ice at a horizontally offset location. The total phytoplankton biomass in the study area ranged between 0.08 μg L^-1^ at a sampling location close to the North Pole (station 370) and 4.5 μg L^-1^ at station 67 in eastern Fram Strait (Fig 3a). In Fram Strait (south of 80°N), Chl *a* concentrations were significantly higher (p = 0.0056, t-test) than in the Central Arctic Ocean (north of 80°N). Values >0.5 μg L^-1^ were observed at most sampling locations in Fram Strait, while total Chl *a* concentrations for the most part did not exceed a concentration of 0.5 μg L^-1^ in the Central Arctic Ocean. This reflects high light availability, lower stratification and better nutrient supply to the surface layer in Fram Strait. In Nansen Basin, low Chl *a* concentrations might be attributed to sea ice coverage, because nutrient concentrations were in a similar range to those observed in eastern Fram Strait. In contrast Chl *a* concentrations in Amundsen Basin were likely limited by low nutrient concentrations since light availability was higher in this area due to the sea ice minimum during the observation period (Table 1). Overall, total Chl *a* values observed in this study reflect a similar situation as observed in summer during a number of previous expeditions into the Central Arctic Ocean around twenty years ago (1993–1999) [40–42]. Back then, summer Chl *a* values were also higher in Fram Strait than in the Central Arctic Ocean and they were in the same range as observed in this study. In a number of different studies during this time period *C*hl *a* values ranged between 0.26 and 0.7 μg L^-1^ in the area of the Nansen- and the Amundsen Basin, while concentrations ~3 μg L^-1^ were observed in the Fram Strait 2014.

### Pico-plankton chlorophyll *a* biomass

Chl *a* biomass of the pico-plankton fraction was also significantly higher in Fram Strait than in the Central Arctic Ocean (p = 0.0044, t-test). The concentrations ranged from 0.048 μg L^-1^ in the Central Arctic Ocean (station 215) to 2.05 μg L^-1^ in eastern Fram Strait (station 67). The average pico-plankton Chl *a* concentration was 0.79 μg L^-1^ in Fram Strait, while it was only 0.16 μg L^-1^ in the Central Arctic Ocean (Fig 3b). In contrast, the contribution of pico-plankton to total Chl *a* biomass was high in both areas (Fig 3c) and did not show significant differences (p = 0.054, t-test). At the majority of sampling locations pico-plankton Chl *a* biomass constituted 60–90% of total Chl *a* biomass. These findings are in agreement with studies from the 1990s that report a contribution of 60–90% of total Chl *a* biomass by small phytoplankton in areas with high ice cover [4, 5]. The relative contribution of pico-plankton biomass was less than 35% at four sampling locations under the influence of continental runoff on the East Greenland Shelf (stations 114, 117, 122) and the Laptev Sea Shelf (311). Total Chl *a* concentrations determined for these locations were higher than average total Chl *a* concentration observed in Polar Waters, but they were lower than the average concentrations determined for the sampling sites in Atlantic Water. Previous studies have emphasized the importance of diatoms associated with the ice edge [43, 44] when ambient light and nutrient concentrations allow a bloom. Thus, it is expected that larger phytoplankton in north Polar Waters contributes significantly to total Chl *a* biomass in the marginal ice zone (MIZ) and in waters influenced by ice melt, e.g., in central Fram Strait and on the Laptev Sea Shelf. We found that the contribution of larger phytoplankton to total Chl *a* biomass was minor in most of our samples, except for the stations located in shelf areas. This might be attributed to the sampling period of our survey, which took place in a post-bloom period during summer at most sampling sites. This was also reflected in the low nutrient concentrations detected at most of the stations sampled. We recognize that the low contribution of larger cells to Chl *a* biomass might be attributed to cell breakage or squeezing of flexible cells during the fractionation process leading to reduced accuracy of sequential filtration. However, a previous study based on sequencing 18S rDNA genes, with focus on pico-eukaryote diversity, supported the accuracy for our sequential filtration approach [3] and we feel confident in the results of our fractionation analysis.

### Biogeographical patterns of pico-eukaryotes in the Arctic Ocean

**Fig 4:**
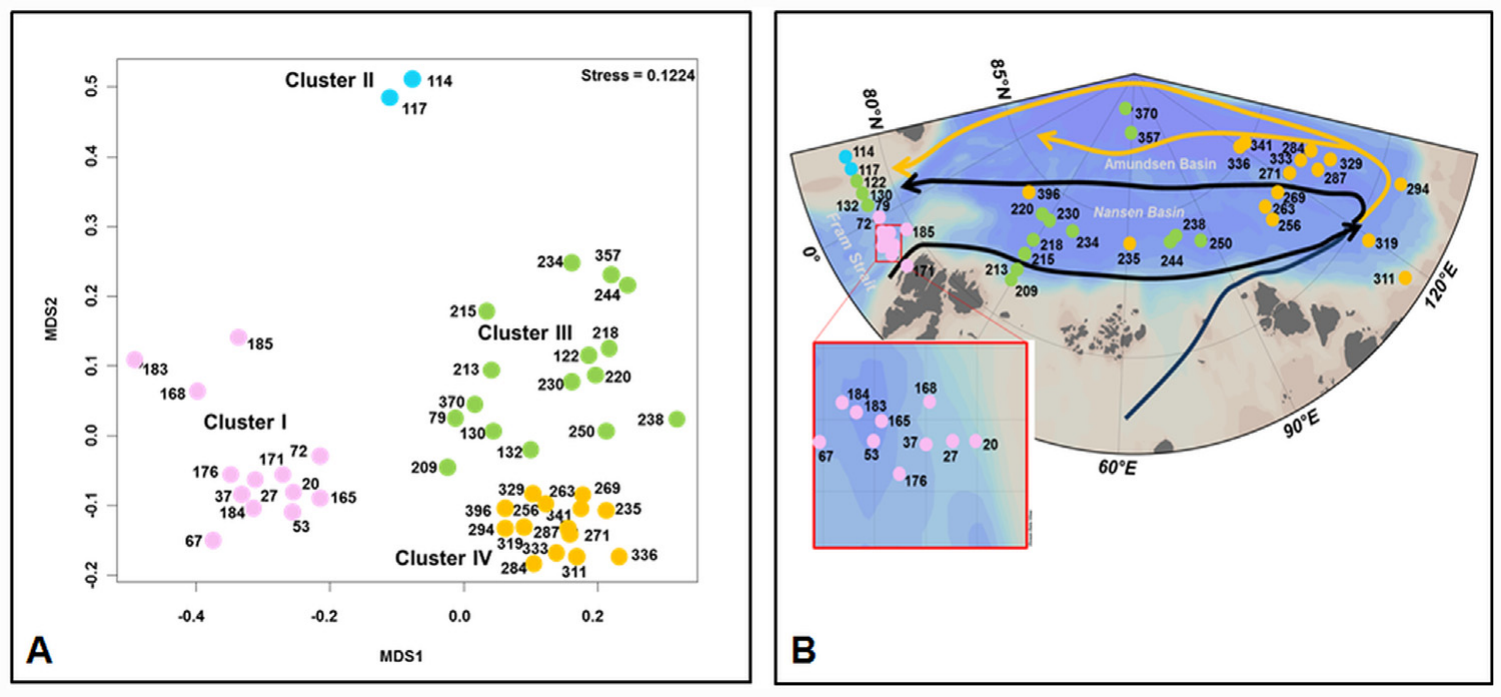
Biogeography of pico-eukaryote communities determined by ARISA. A: Meta MDS plot displaying similarity between pico-eukaryote communities based on the Jaccard Index. B: Allocation of grouping in the MDS-plot to sampling locations. Modified after Rudels et al. (2012), major ocean currents were sketched into the map. Atlantic inflow is sketched in black, while modified Atlantic Water is sketched in orange.

Biogeographical patterns of Arctic pico-eukaryote communities were determined using ARISA. This fingerprinting method is a quick and cost-efficient method that allows processing of high numbers of samples. Its explanatory power to elucidate variability in protist community composition is high, as the fingerprint profiles reflect variability in community composition very accurately [45]. ARISA was run on the genomic DNA isolated from the Chl *a* maximum of 46 sampling locations. Based on the Jaccard’s distances the ARISA profiles were grouped *à priori* into four distinct clusters in a metaMDS-plot (Fig 4a), and the clustering is supported by an ANOSIM (R = 0.72, p = 0.001). This clustering is similar to the clustering of ambient salinity, water temperature and ice cover at the time and location of the sampling reflecting in part the contrast between Cluster I in Atlantic Water and Cluster IV in generally fresher waters than in Cluster III (Fig 2). The similarity is statistically supported by a Mantel-test (r = 0.49, p = 0.001). Cluster I contains ARISA profiles derived from samples collected in Atlantic Water (Fig 4b). Cluster II contains ARISA profiles obtained on the Greenland Shelf from samples collected in Polar Water modified by continental runoff from Greenland. The ARISA profiles grouping in Cluster III mainly represent sampling locations in Polar Waters of western Fram Strait and in the Nansen Basin. Finally, Cluster IV mainly contains ARISA profiles of samples collected in the Amundsen Basin. Several other studies have suggested that phytoplankton diversity and activity in the Arctic may be water mass specific [18, 46]. Consistent with this, the clustering of the molecular fingerprint patterns in this study is best explained in relation to ambient water mass characteristics. The molecular fingerprints from samples collected in western Fram Strait and Nansen Basin clustered together. We propose that similarities and dissimilarities between molecular fingerprints originating from samples collected in the Arctic Ocean might be explained by ocean currents in the Arctic Ocean that achieve connectivity between Arctic marine microbial communities (Fig 4b). In the Nansen Basin and over the Gakkel Ridge, the Transpolar Drift carries more saline halocline waters originating from the Fram Strait inflow branch back to the Fram Strait. Conversely, the halocline waters in the Amundsen Basin have lower salinity. In our study we observed similar molecular fingerprinting patterns in the Nansen Basin and the western Fram Strait. Pico-eukaryotes endemic in cooled Atlantic Water might be transported via the return flow from Nansen Basin to western Fram Strait. The other water masses display differences in their fingerprinting profiles because of significant differences in environmental parameters. The Atlantic Water inflow in eastern Fram Strait is warm and saline and has comparatively low stratification conducive to deeper mixed layers and nutrient supply from below the euphotic zone. These waters carry a distinct pico-eukaryote community reflected by a distinct molecular fingerprint deduced from the samples collected in this area. Pico-eukaryotes endemic in these waters are probably transported into the Nansen Basin via the West Spitsbergen Current, where they finally disappear, despite similar nutrient availability in the area. This could be attributed to lower water temperatures and increased stratification associated with the halocline formation or to the sea ice cover present there. We cannot exclude that seasonal effects contribute to differences in the molecular fingerprint patterns of the different water masses, but we think that they are of minor importance. This is because samples with similar fingerprint patterns were collected with a time-lag of two months (samples from western Fram Strait and Nansen Basin), while other samples with significantly distinct fingerprint patterns were collected with a shorter time-lag of only a few days (samples from Nansen Basin and Amundsen Basin).

### Community composition

In order to elucidate the taxonomic composition accounting for differences in ARISA fragment composition, we ran 454-pyrosequencing of the 18S rDNA V4 region on a subset of 17 samples from the <3 μm fraction. The samples were chosen according to their clustering in the metaMDS-plot and for differences in environmental conditions or geographical location. The subset of pico-eukaryotic samples is composed of samples that allow comparison of the community composition in Atlantic Water versus. Polar Water, at shelf stations versus deep-sea areas, in the transition zone between Atlantic Water and Polar Water in Fram Strait, and in the Nansen Basin versus Amundsen Basin. This approach provides taxonomic insight into environment related and biogeographical patterns in the structure of Arctic pico-eukaryote communities. Variability in qualitative species composition observed by 454-pyrosequencing confirmed the results of the ARISA-profiling. Based on Jaccard’s distances, grouping of 454-sequence libraries was highly similar to the grouping of the ARISA profiles (Mantel test: R = 0.7179, p = 0.001).

**Fig 5:**
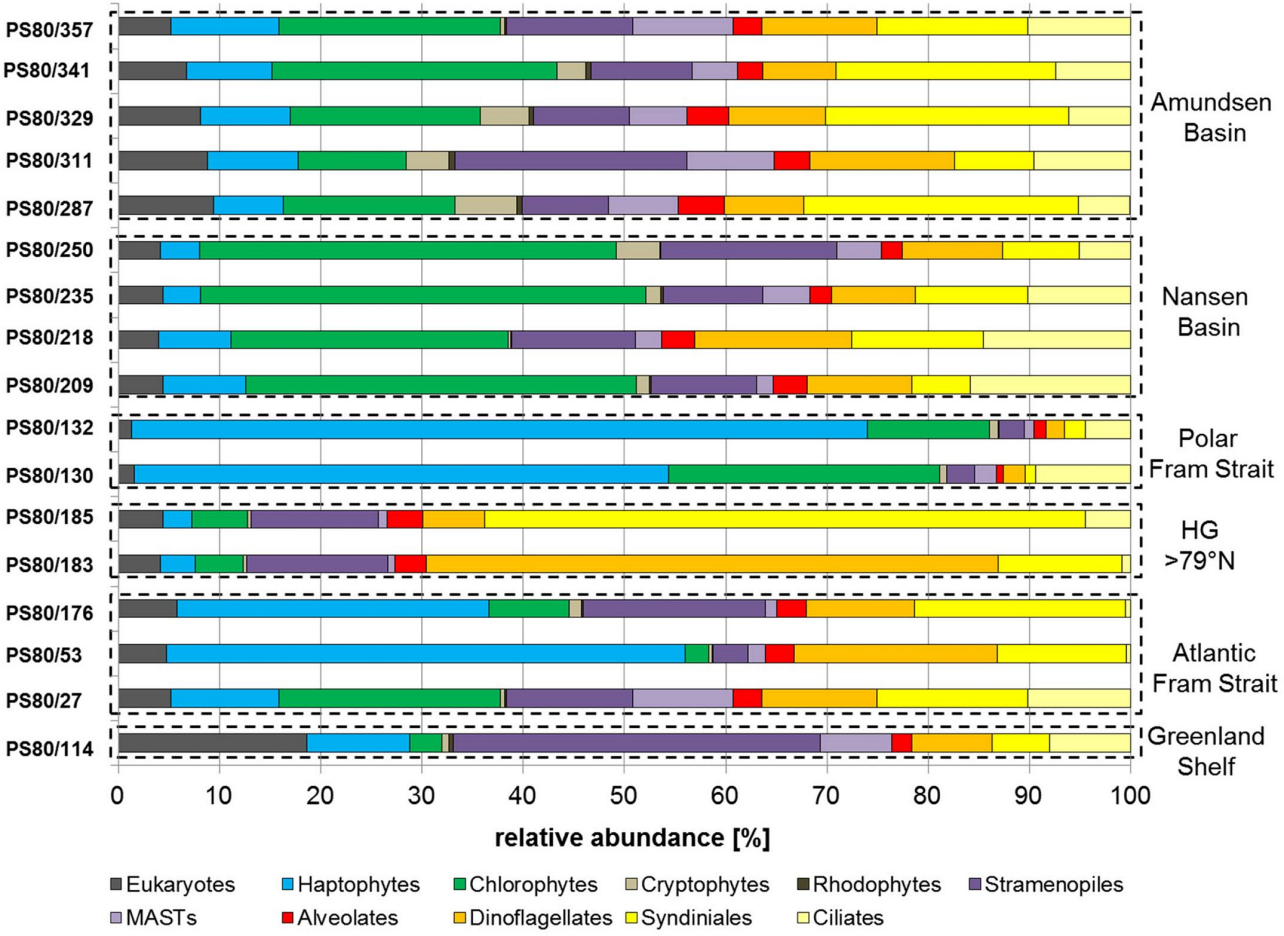
Community assemblage based on 454-NGS sequencing of selected samples representing the grouping in the MetaMDS-plot.

**Fig 6:**
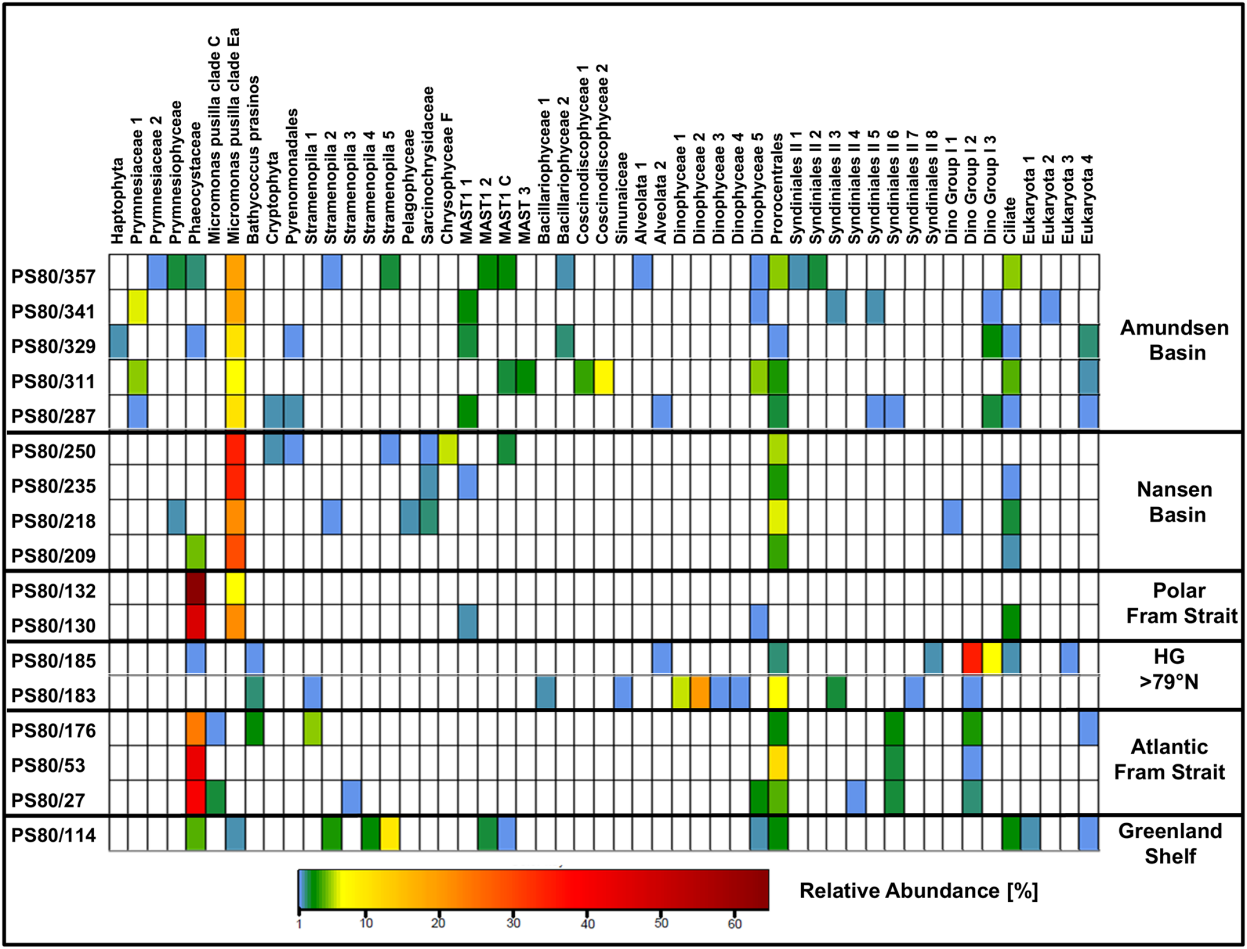
Assemblage of the abundant biosphere, representing operational taxonomic units (OUTs) that constitute >1% of sequences in a sample. An OTU represents a cluster of sequences with 97% similarity in the sequence of the 18S rRNA V4 region. The numbering of taxa reflects different sequences that fall into this branch of the phylogenetic tree, but that could not be annotated with higher taxonomic resolution.

Two out of three pico-eukaryote samples collected in Atlantic Water at stations along a transect on 78°50 N (stations 53, 176) were dominated by sequences affiliating in the phylogenetic tree with haptophytes (Fig 5). These two stations displayed a similar community structure even though sampling at station 176 took place around four weeks after the sampling at station 53. At station 27, also collected in Atlantic Water, the read composition was slightly different. Here, chlorophytes contributed a higher share of all sequence reads, but the abundant biosphere (>1% of sequence reads) of all three samples was dominated by sequences annotated as Phaeocystaceae (haptophytes). They contributed more than 40% of abundant reads in all samples collected in Atlantic Waters in Fram Strait south of 79°N (Fig 6). Other sequences of the abundant biosphere in these samples were annotated as Prorocentrales, Syndiniales or Mamiellales. The pico-eukaryote communities collected in Atlantic Water north of 79°N (stations 183,185) were dominated by sequences annotated as Dinophyceae and Syndiniales (>60% of sequence reads), while haptophytes or chlorophytes accounted for less than 10% of all sequence reads (Fig 5). Station 185 was nitrate depleted (0.17 μmol L^-1^), which might explain the high proportion of potentially mixotrophic or heterotrophic dinoflagellates. The pico-eukaryote communities collected in open oceanic Polar Waters of Fram Strait on 78°50’ (stations 130, 132) displayed a similar 18S rDNA based community structure as observed in samples collected in Atlantic Water of Fram Strait. The communities at these stations were also clearly dominated by sequences annotated as haptophytes (Fig 5). Most sequences were contributed by the same strain of Phaeocystaceae (>60% of the abundant biosphere) that was also observed in the Atlantic Water of Fram Strait (Fig 6). In contrast to the observations for Polar Waters in Fram Strait, the share of 18S rDNA reads related to haptophytes never exceeded 10% of total read number in the waters of the Central Arctic Ocean (Fig 5). The contribution of haptophytes to the pico-eukaryote community was slightly lower in Nansen Basin than in Amundsen Basin. The dominant Phaeocystaceae observed in Fram Strait was mainly found in the rare biosphere (<1% of total reads) of samples collected in the Central Arctic Ocean. Phaeocystaceae sequence abundance was positively correlated with Chl *a* biomass. A Pearson’s product-moment correlation revealed that correlation between Phaeocystaceae sequence abundance and total Chl *a* was 72%, respectively 90% with pico-eukaryote Chl *a*. These results suggest that this family is a major contributor to Chl *a* biomass in the study area. A rapid increase of single celled *Phaeocystis pouchetii* (Hariot) Lagerheim during sea ice retreat was reported previously around Svalbard [47]. In our study the dominance of reads annotated as Phaeocystaceae in the pico-eukaryote fraction indicated the presence of single cells. Single cells of the genus *Phaeocystis* sp. have been documented to have a size of ~3 μm [48] and may have passed the filter used to collect the pico-plankton fraction. Another study reports that *P*. *pouchetii*, present mainly in its colonial form represented more than 90% of total phytoplankton bio-volume in Fram Strait in July 2007 [49]. Currently, knowledge on mechanisms or environmental conditions that trigger colony formation of *P*. *pouchetii* is scarce [50]. North of 80°N (Nansen Basin and Amundsen Basin) the contribution of Phaeocystaceae to the sequence assemblage was maximum ~7%. Moreover, the OTU dominating sequence assemblages south of 80°N was not present in the abundant biosphere of most samples north of 79°N. South of 80°N, however, high abundances of Phaeocystaceae sequences were observed in both warm ice-free Atlantic Water and ice-covered Polar Water of Fram Strait. This suggests either the potential of this species to “bloom” in cold ice-covered water or advection of Phaeocystaceae under the ice, possibly due to a short advection pathway in the Atlantic Water recirculating in Fram Strait [51]. At much larger advection pathways we observed very low abundances of Phaeocystaceae in the ice covered regions of Nansen Basin and Amundsen Basin.

In the Central Arctic Ocean, chlorophytes dominated the pico-eukaryote communities (Fig 5). At the sampling locations in the open ocean area of Nansen Basin (stations 218, 235, 250, 357) the sequences assigned as Chlorophyta contributed ~40% of all sequence reads generated from these samples, while in the waters of the Amundsen Basin (stations 287, 329 and 341) the contribution of Chlorophyta sequences was around 25%. The share of reads annotated as Mamiellaceae (Chlorophyta) at sampling locations in Fram Strait was lower than in the Central Arctic Ocean. In Fram Strait, contribution of Mamiellaceae to the abundant biosphere never exceeded 20%, while they contributed >50% of sequence reads in the area of Nansen Basin and 20–30% in Amundsen Basin. Furthermore, we observed different strains of Mamiellaceae in the abundant biosphere of Polar and Atlantic Water. Ecotype *Micromonas pusilla* (Butcher) Manton&Parke Clade Ea (Mamiellaceae) isolated in Canadian Arctic Waters [15] was only found in the abundant biosphere of Polar Waters, while ecotype *M*. *pusilla* Clade C [6] was only found in the abundant biosphere of Atlantic Water (Fig 6). *Micromonas pusilla* constitutes high shares of sequences at the Central Arctic sites, but they were significantly higher in Nansen Basin (~40%) than in Amundsen Basin (~10–20%). In this study, salinity in Nansen Basin was intermediate (~33–34) compared to eastern Fram Strait and Amundsen Basin. Previous studies on Arctic pico-eukaryote diversity reported a dominance of the Arctic ecotype *M*. *pusilla* CCMP 2099 (Mamiellaceae) in cold waters, with high ice concentration and intermediate salinity [46, 52]. Recently, light and nutrient determined phagotrophy has been shown for the Arctic ecotype of *M*. *pusilla* (CCMP 2099) and bacteria are the preferential food source. Ingestion rates were highest under low-nutrient and high-light conditions [53]. The Arctic ecotype of *M*. *pusilla* grows fast with low light and at low temperatures [15]. Overall, the Arctic strain of *M*. *pusilla* seems to be well adapted to growth under the harsh conditions in the Central Arctic Ocean. Phagotrophy might insure survival under nutrient-limited conditions, while adaptation to low-light conditions might insure survival under the ice.

Besides Phaeocystaceae and *M*. *pusilla*, small dinoflagellates were major constituents of our Arctic protist communities in summer 2012 in the Central Arctic Ocean as well as in Fram Strait. They contributed ~25–40% of all sequence reads south of 79°N. This is also in agreement with previous studies that reported a contribution of 30–40% of these taxa to total phytoplankton biomass south of 80°N [44, 54]. Data on phytoplankton abundance and community structure north of 80°N are scarce due to the persistence of sea ice during summer and our data may constitute relevant new information on pico-eukaryote biogeography in this area. In eastern Fram Strait, Nansen Basin, Amundsen Basin and over the Greenland Shelf, dinoflagellate and Syndiniales sequences contributed equally to total sequence assemblage (~10%). Prorocentrales contributed similarly to the total sequence assemblage of all samples in this study. However, in western Fram Strait the share of dinoflagellates was very low, while it was particularly high in eastern Fram Strait north of 79°N. In our study, sequences related to small flagellates and dinoflagellates contributed >60% of all sequences derived from phytoplankton communities collected in Fram Strait north of 79°N. The proportion of dinoflagellates and Syndiniales was inversely correlated. The contribution of sequence reads affiliated with dinoflagellates and syndiniales was higher in Amundsen Basin than in Nansen Basin. Syndiniales contributed exclusively to the abundant biosphere of Amundsen Basin. Syndiniales are a dinoflagellate group composed exclusively of marine parasites [55]. Syndiniales Group II sequences were found in this study in the abundant biosphere of samples collected in ice-free Atlantic Waters and in Amundsen Basin, suggesting that Syndiniales Group II prefer waters with higher light availability. This assumption is supported by previous studies based on clone library sequencing that report a dominance of Syndiniales Group II sequences in sunlit marine surface waters [56]. In contrast, many dinoflagellates are mixotroph or heterotroph [57], which might be a competitive advantage compared to Syndiniales under low light conditions in nutrient limited ice covered regions of the Arctic Ocean.

## Conclusions

Our data suggest that Chl *a* concentrations and the contribution of pico-plankton to pelagic Chl *a* biomass were not significantly affected by the sea ice minimum in 2012 since the concenctrations observed in this study were in a similar range as those observed around 20 years ago in the observation area. Distribution of Chl *a* biomass and biogeographic patterns of pico-eukaryote communities were best understood in relation to ambient water mass characteristics and sea ice coverage. Pico-eukaryote community composition and biogeography in the Arctic Ocean is probably a result of advection of taxa by oceanic currents that also impact nutrient distribution. Due to the Atlantic inflow, nutrient concentrations in Nansen Basin were similar to those observed in eastern Fram Strait, but ice coverage was higher in Nansen Basin than in Fram Strait. This leaves scope to speculate that pico-eukaryote community composition in the current halocline formation area of Nansen Basin could shift towards the situation currently observed in eastern Fram Strait if sea ice retreat progresses in the future and nutrient concentrations remain at least in the range observed in this study. This could increase Chl *a* biomass in the area of the Nansen Basin and consequently strongly affect carbon cycles in the area.

## Supporting Information

S1 File R-script of the “Interactive Binner” that was used to generate presence/absence matrices reflecting the community profiles of the samples by binning the quality controlled data obtained after size separation with the capillary sequencer. (DOCX)

S2 File R-script that was used to visualize grouping of samples based on differences in fragment composition by multidimensional scaling (MDS). Groups in the MDS plot were determined *à priori* based on automated clustering using the hclust function in R.
(TXT)

## References

[1] Vaulot D, Eikrem W, Viprey M, Moreau H. The diversity of small eukaryotic phytoplankton (< = 3μm) in marine ecosystems. Fems Microbiol Rev. 2008;32(5):795–820. 10.1111/j.1574-6976.2008.00121.x WOS:000258402300003. 18564290

[2] Sherr EB, Sherr BF, Wheeler PA, Thompson K. Temporal and spatial variation in stocks of autotrophic and heterotrophic microbes in the upper water column of the central Arctic Ocean. Deep-Sea Res Part I-Oceanogr Res Pap. 2003;50(5):557–71. WOS:000188458300001.

[3] Kilias ES, Noethig E-M, Wolf C, Metfies K. Picoeukaryote Plankton Composition off West Spitsbergen at the Entrance to the Arctic Ocean. J Eukaryot Microbiol. 2014;61(6):569–79. 10.1111/jeu.12134 WOS:000344645600002. 24996010

[4] Gosselin M, Levasseur M, Wheeler PA, Horner RA, Booth BC. New measurements of phytoplankton and ice algal production in the Arctic Ocean. Deep-Sea Res Part II-Top Stud Oceanogr. 1997;44(8):1623-+. WOS:000072772400009.

[5] Booth BC, Horner RA. Microalgae on the Arctic Ocean Section, 1994: species abundance and biomass. Deep-Sea Res Part II-Top Stud Oceanogr. 1997;44(8):1607–22. WOS:000072772400008.

[6] Slapeta J, Lopez-Garcia P, Moreira D. Global dispersal and ancient cryptic species in the smallest marine eukaryotes. Mol Biol Evol. 2006;23(1):23–9. 10.1093/molbev/msj001 ISI:000233843900005. 16120798

[7] Hartmann DL, Klein Tank AMG, Rusticucci M, Alexander LV, Brönnimann S, Charabi Y, et al Observations: Atmosphere and Surface. In: Climate Change 2013: The Physical Science Basis Contribution of Working Group I to the Fifth Assessment Report of the Intergovernmental Panel on Climate Change [Stocker TF, Qin D, Plattner G-K, Tignor M, Allen SK, Boschung J, Nauels A, Xia Y, Bex V and Midgley PM (eds)] Cambridge University Press, Cambridge, United Kingdom and New York, NY, USA 2013:159–254.

[8] Kwok R, Untersteiner N. The thinning of Arctic sea ice. Phys Today. 2011;64(4):36–41. WOS:000289397900015.

[9] Perovich DK. The changing Arctic sea ice cover. Oceanography. 2011;24(3):162–73. WOS:000295394700023.

[10] Nicolaus M, Katlein C, Maslanik J, Hendricks S. Changes in Arctic sea ice result in increasing light transmittance and absorption. Geophys Res Lett. 2012;39 L24501 10.1029/2012gl053738 WOS:000312943500001.

[11] Rabe B, Karcher M, Kauker F, Schauer U, Toole JM, Krishfield RA, et al Arctic Ocean basin liquid freshwater storage trend 1992–2012. Geophys Res Lett. 2014;41(3):961–8. 10.1002/2013gl058121 WOS:000332990500031.

[12] Arrigo KR, Perovich DK, Pickart RS, Brown ZW, van Dijken GL, Lowry KE, et al Massive Phytoplankton Blooms Under Arctic Sea Ice. Science. 2012;336(6087):1408- 10.1126/science.1215065 WOS:000305211700035. 22678359

[13] Moran XAG, Lopez-Urrutia A, Calvo-Diaz A, Li WKW. Increasing importance of small phytoplankton in a warmer ocean. Glob Change Biol. 2010;16(3):1137–44. 10.1111/j.1365-2486.2009.01960.x ISI:000274419500018.

[14] Sun S, Bleck R. Geographic distribution of the diapycnal component of thermohaline circulations in coupled climate models. Ocean Modelling. 2006;15(3–4):177–99. WOS:000242740800004.

[15] Lovejoy C, Vincent WF, Bonilla S, Roy S, Martineau MJ, Terrado R, et al Distribution, phylogeny, and growth of cold-adapted picoprasinophytes in arctic seas. J Phycol. 2007;43(1):78–89. 10.1111/j.1529-8817.2006.00310.x ISI:000244004300009.

[16] Lovejoy C, Potvin M. Microbial eukaryotic distribution in a dynamic Beaufort Sea and the Arctic Ocean. J Plankton Res. 2011;33(3):431–44. 10.1093/plankt/fbq124 WOS:000287025500007.

[17] Comeau AM, Li WKW, Tremblay JE, Carmack EC, Lovejoy C. Arctic Ocean Microbial Community Structure before and after the 2007 Record Sea Ice Minimum. PLoS One. 2011;6(11). e2749210. WOS:000297350800055. 10.1371/journal.pone.0027492 PMC3212577 22096583

[18] Hamilton AK, Lovejoy C, Galand PE, Ingram RG. Water masses and biogeography of picoeukaryote assemblages in a cold hydrographically complex system. Limnology and Oceanography. 2008;53(3):922–35. 10.4319/lo.2008.53.3.0922 WOS:000256498900005.

[19] Parkinson CL, Comiso JC. On the 2012 record low Arctic sea ice cover: Combined impact of preconditioning and an August storm. Geophys Res Lett. 2013;40(7). 10.1002/grl.50349 WOS:000319217600019.

[20] L. E. Recommendations on methods for marine biological studies in the Baltic Sea. Phytoplankton and chlorophyll. Marine Biologists Publication. 1979;5.

[21] Evans CAOR, J. E. A manual for measurement of chlorophyll a in netplankton and nanoplankton. Ocean Pulse Technical Manual. 1980;3:SHRL80–17.

[22] Medlin L, Elwood HJ, Stickel S, Sogin ML. The characterization of enzymatically amplified eukaryotic 16S-like rRNA -coding regions. Gene. 1988;71(2):491–9. WOS:A1988R610900027. 3224833 10.1016/0378-1119(88)90066-2

[23] TJ W, T B, S L, J T. Amplification and direct sequencing of fungal ribosomal RNA genes for phylogenetics. PCR Protoc: Guide Methods Appl. 1990;18 315–22

[24] Ramette A. Quantitative Community Fingerprinting Methods for Estimating the Abundance of Operational Taxonomic Units in Natural Microbial Communities. Appl Environ Microbiol. 2009;75(8):2495–505. 10.1128/aem.02409-08 WOS:000264936800030. 19201961 PMC2675222

[25] Jaccard P. The distribution of flora in the Alpine Zone.1. New Phytologist. 1912;11(2):37–50. 10.1111/j.1469-8137.1912.tb05611.x

[26] Clarke KR. Non-parametric multivariate analysis of changes in community structure. Australian Journal of Ecology. 1993;18:117–43.

[27] Elwood HJ, Olsen GJ, Sogin ML. The small-subunit ribosomal RNA gene-sequence from the hypohrichous ciliates Oxytricha-nova and Stylonychia-pustulata. Molecular Biology and Evolution. 1985;2(5):399–410. WOS:A1985ARH2000004. 3939705 10.1093/oxfordjournals.molbev.a040362

[28] Nickrent DL, Sargent ML. An overview of the secondary structure of the V$-region of eukaryotic small-subunit ribosomal-RNA. Nucleic Acids Research. 1991;19(2):227–35. ISI:A1991EV86800003. 2014163 10.1093/nar/19.2.227 PMC333584

[29] Edgar RC, Haas BJ, Clemente JC, Quince C, Knight R. UCHIME improves sensitivity and speed of chimera detection. Bioinformatics. 2011;27(16):2194–200. 10.1093/bioinformatics/btr381 WOS:000293620800004. 21700674 PMC3150044

[30] Behnke A, Engel M, Christen R, Nebel M, Klein RR, Stoeck T. Depicting more accurate pictures of protistan community complexity using pyrosequencing of hypervariable SSU rRNA gene regions. Environ Microbiol. 2011;13(2):340–9. 10.1111/j.1462-2920.2010.02332.x WOS:000286835900006. 21281421

[31] Kunin V, Engelbrektson A, Ochman H, Hugenholtz P. Wrinkles in the rare biosphere: pyrosequencing errors can lead to artificial inflation of diversity estimates. Environ Microbiol. 2010;12(1):118–23. 10.1111/j.1462-2920.2009.02051.x WOS:000274234200011. 19725865

[32] Miranda LN, Zhuang YY, Zhang H, Lin S. Phylogenetic analysis guided by intragenomic SSU rDNA polymorphism refines classification of "Alexandrium tamarense" species complex. Harmful Algae. 2012;16:35–48. WOS:000303298600005.

[33] Eddy SR. Accelerated Profile HMM Searches. PLoS Comput Biol. 2011;7(10). e100219510. WOS:000297262700026. 10.1371/journal.pcbi.1002195 PMC3197634 22039361

[34] Matsen FA, Kodner RB, Armbrust EV. pplacer: linear time maximum-likelihood and Bayesian phylogenetic placement of sequences onto a fixed reference tree. Bmc Bioinformatics. 2010;11. 53810.1186/1471-2105-11-538. WOS:000284533800001. 10.1186/1471-2105-11-538 PMC3098090 21034504

[35] Aksenov Y, Ivanov VV, Nurser AJG, Bacon S, Polyakov IV, Coward AC, et al The Arctic Circumpolar Boundary Current. J Geophys Res-Oceans. 2011;116. C0901710.1029/2010jc006637. WOS:000295132800002.

[36] Rudels B, Bjork G, Nilsson J, Winsor P, Lake I, Nohr C. The interaction between waters from the Arctic Ocean and the Nordic Seas north of Fram Strait and along the East Greenland Current: results from the Arctic Ocean-02 Oden expedition. J Mar Syst. 2005;55(1–2):1–30. WOS:000228346400001.

[37] de Steur L, Hansen E, Gerdes R, Karcher M, Fahrbach E, Holfort J. Freshwater fluxes in the East Greenland Current: A decade of observations. Geophys Res Lett. 2009;36. L2361110.1029/2009gl041278. WOS:000272941100002.

[38] Jones EP, Anderson LG, Swift JH. Distribution of Atlantic and Pacific waters in the upper Arctic Ocean: Implications for circulation. Geophys Res Lett. 1998;25(6):765–8. 10.1029/98gl00464 WOS:000072645900002.

[39] Beszczynska-Moller A, Fahrbach E, Schauer U, Hansen E. Variability in Atlantic water temperature and transport at the entrance to the Arctic Ocean, 1997–2010. ICES J Mar Sci. 2012;69(5):852–63. 10.1093/icesjms/fss056 WOS:000305460200019.

[40] Cai P, van der Loeff MR, Stimac I, Nothig EM, Lepore K, Moran SB. Low export flux of particulate organic carbon in the central Arctic Ocean as revealed by Th-234:U-238 disequilibrium. J Geophys Res-Oceans. 2010;115. C1003710.1029/2009jc005595. WOS:000283092300001.

[41] Damm E, Helmke E, Thoms S, Schauer U, Nothig E, Bakker K, et al Methane production in aerobic oligotrophic surface water in the central Arctic Ocean. Biogeosciences. 2010;7(3):1099–108. WOS:000276180300020.

[42] Cherkasheva A, Bracher A, Melsheimer C, Koeberle C, Gerdes R, Noethig EM, et al Influence of the physical environment on polar phytoplankton blooms: A case study in the Fram Strait. J Mar Syst. 2014;132:196–207. WOS:000334141400017.

[43] Falk-Petersen S, Sargent JR, Henderson J, Hegseth EN, Hop H, Okolodkov YB. Lipids and fatty acids in ice algae and phytoplankton from the Marginal Ice Zone in the Barents Sea. Polar Biology. 1998;20(1):41–7. 10.1007/s003000050274 WOS:000074814400005.

[44] Richardson K, Markager S, Buch E, Lassen MF, Kristensen AS. Seasonal distribution of primary production, phytoplankton biomass and size distribution in the Greenland Sea. Deep-Sea Res Part I-Oceanogr Res Pap. 2005;52(6):979–99. WOS:000229525800005.

[45] Kilias ES, Wolf C, Metfies K. Characterizing variability in marine protist communities via ARISA fingerprints—a method evaluation. Limnology and Oceanography: Methods. 2015;13(2):74–80. 10.1002/lom3.10008

[46] Kilias E, Kattner G, Wolf C, Frickenhaus S, Metfies K. A molecular survey of protist diversity through the central Arctic Ocean. Polar Biology. 2014;37(9):1271–87. 10.1007/s00300-014-1519-5 WOS:000339893400005.

[47] Wassmann P, Ratkova T, Reigstad M. The contribution of single and colonial cells of Phaeocystis pouchetii to spring and summer blooms in the north-eastern North Atlantic. Harmful Algae. 2005;4(5):823–40. WOS:000231256200003.

[48] Gaebler-Schwarz S, Davidson A, Assmy P, Chen JX, Henjes J, Nothig EM, et al A new cell stage in the haploid-diploid life cycle of the colony-forming haptophyte Phaeocystis antarctica and its ecological implications. J Phycol. 2010;46(5):1006–16. 10.1111/j.1529-8817.2010.00875.x WOS:000282378200018.

[49] Lasternas S, Agusti S. Phytoplankton community structure during the record Arctic ice-melting of summer 2007. Polar Biology. 2010;33(12):1709–17. 10.1007/s00300-010-0877-x WOS:000286392500011.

[50] Peperzak L, Gabler-Schwarz S. Current knowledge of the life cycles of Phaeocystis globosa and Phaeocystis antarctica (Prymnesiophyceae). J Phycol. 2012;48(3):514–7. 10.1111/j.1529-8817.2012.01136.x WOS:000304810200002. 27011066

[51] de Steur L, Hansen E, Mauritzen C, Beszczynska-Moeller A, Fahrbach E. Impact of recirculation on the East Greenland Current in Fram Strait: Results from moored current meter measurements between 1997 and 2009. Deep-Sea Res Part I-Oceanogr Res Pap. 2014;92:26–40. WOS:000342260200003.

[52] Kilias E, Wolf C, Noethig E-M, Peeken I, Metfies K. Protist distribution in the western Fram Strait based on 454-Pyrosequencing of 18S rDNA. J Phycol. 2013;49(5):996–1010. 10.1111/jpy.12109 WOS:000327899700016. 27007321

[53] McKie-Krisberg ZM, Sanders RW. Phagotrophy by the picoeukaryotic green alga Micromonas: implications for Arctic Oceans. Isme J. 2014;8(10):1953–61. 10.1038/ismej.2014.16 WOS:000342764600001. 24553471 PMC4184008

[54] Rat'kova TN, Wassmann P. Seasonal variation and spatial distribution of phyto- and protozooplankton in the central Barents Sea. J Mar Syst. 2002;38(1–2):47–75. WOS:000179499900004.

[55] Moon-van der Staay SY, De Wachter R, Vaulot D. Oceanic 18S rDNA sequences from picoplankton reveal unsuspected eukaryotic diversity. Nature. 2001;409(6820):607–10. 10.1038/35054541 WOS:000166692300041. 11214317

[56] Guillou L, Viprey M, Chambouvet A, Welsh RM, Kirkham AR, Massana R, et al Widespread occurrence and genetic diversity of marine parasitoids belonging to Syndiniales (Alveolata). Environ Microbiol. 2008;10(12):3349–65. 10.1111/j.1462-2920.2008.01731.x WOS:000260744800016. 18771501

[57] Jeong HJ, Yoo YD, Kim JS, Seong KA, Kang NS, Kim TH. Growth, Feeding and Ecological Roles of the Mixotrophic and Heterotrophic Dinoflagellates in Marine Planktonic Food Webs. Ocean Science Journal. 2010;45(2):65–91. KJD:ART001456060.

