# Supplementary material for: Biogeography and Photosynthetic Biomass of Arctic Marine Pico-Eukaroytes during Summer of the Record Sea Ice Minimum 2012
Source: PLoS One. 2016 Feb 19;11(2):e0148512. doi: 10.1371/journal.pone.0148512 (PMC4760976; doi:10.1371/journal.pone.0148512)
Supplement: S1 File — (DOCX) [file pone.0148512.s001.docx]

# version 1.3

# Copyright (C) 2008 Alban Ramette

# This program is free software; you can redistribute it and/or modify

# it under the terms of the GNU General Public License as published by

# the Free Software Foundation; either version 2 of the License, or

# (at your option) any later version.

#

# This program is distributed in the hope that it will be useful,

# but WITHOUT ANY WARRANTY; without even the implied warranty of

# MERCHANTABILITY or FITNESS FOR A PARTICULAR PURPOSE. See the

# GNU General Public License for more details.

automaticbinner=function(D){

cat("----------------Automatic binner v.1.3. by A. Ramette----------------\n")

cat('NOTE: The user needs to set the working directory, as follows:\n')

cat(' for instance: setwd("c:\\\\R\\\\DIR")\n')

cat(' The result files will be saved in that DIR directory\n')

cat('NOTE: The user needs to import the D table before starting\n')

cat(" e.g. D=read.table(\"input.txt\",h=TRUE)\n")

cat(" D is a table with 3 columns:\n")

cat(" D[,1] sample name for each band\n")

cat(" D[,2] the second consists of band sizes\n")

cat(" D[,3] the last consists of area (fluorescence in absolute value\n")

cat(" Automatic mode... \n")

cat("------------------------------------------------------------\n")

ANS<-readline("Continue? (y/n)....... ")

if(ANS=="y"){

###################### variable declaration

Sm=as.numeric(readline( "\nLower bound of the size range, e.g. 100:\t"))

SM=as.numeric(readline( "Higher bound of the size range, e.g. 1000:\t"))

RFIco=as.numeric(readline( "Minimum RFI cutoff value, e.g. 0.09%:\t\t"))
